# Single-cell biomagnifier for optical nanoscopes and nanotweezers

**DOI:** 10.1038/s41377-019-0168-4

**Published:** 2019-07-03

**Authors:** Yuchao Li, Xiaoshuai Liu, Baojun Li

**Affiliations:** Institute of Nanophotonics, Jinan University, 511443 Guangzhou, China

**Keywords:** Optical manipulation and tweezers, Imaging and sensing

## Abstract

Optical microscopes and optical tweezers, which were invented to image and manipulate microscale objects, have revolutionized cellular and molecular biology. However, the optical resolution is hampered by the diffraction limit; thus, optical microscopes and optical tweezers cannot be directly used to image and manipulate nano-objects. The emerging plasmonic/photonic nanoscopes and nanotweezers can achieve nanometer resolution, but the high-index material structures will easily cause mechanical and photothermal damage to biospecimens. Here, we demonstrate subdiffraction-limit imaging and manipulation of nano-objects by a noninvasive device that was constructed by trapping a cell on a fiber tip. The trapped cell, acting as a biomagnifier, could magnify nanostructures with a resolution of 100 nm (*λ*/5.5) under white-light microscopy. The focus of the biomagnifier formed a nano-optical trap that allowed precise manipulation of an individual nanoparticle with a radius of 50 nm. This biomagnifier provides a high-precision tool for optical imaging, sensing, and assembly of bionanomaterials.

## Subwavelength imaging: Seeing smaller through cells

An optical microscope system that uses living cells as tiny lenses to image and manipulate objects smaller than the wavelength of light has been demonstrated by researchers in China. Optical microscopes and laser tweezers can image and trap objects as small as a few hundred nanometers, but it is difficult to apply these tools to anything smaller than half the wavelength of light because of the so-called diffraction limit. Yuchao Li and co-workers at Jinan University demonstrated that bacteria, yeast, red blood cells or stem cells positioned beneath a microscope can focus light to resolve details smaller than a fifth of the wavelength, such as the fibrous cytoskeleton in larger cells. The team also used the subwavelength light spot focused by the “biomagnifier” cells to trap and manipulate nanoparticles just 50 nanometers in radius.

## Introduction

Optical imaging and manipulation of small objects is crucial in the fields of medical diagnosis^1,2^, biological sensing^3,4^, cellular exploration^5,6^, molecular tracking^7,8^, and material assembly^9,10^. Optical tweezers and optical microscopes have become standard devices for noncontact imaging and manipulation of samples ranging in diameters from a few hundreds of nanometers to several tens of micrometers^11–13^. However, it is challenging to apply these tools to nanoscale objects (0–100 nm) because the optical resolution is restricted to around half of the illumination wavelength by the far-field diffraction barrier^14,15^. The past few decades have witnessed dramatic progress of near-field nanoscopes and nanotweezers that achieve optical imaging and manipulation with nanometer resolution^16,17^. Near-field nanoscopes and nanotweezers, such as optical nanoprobes^18^, superlenses^19^, plasmonic tweezers^20^, and photonic crystal tweezers^21^, are generally driven by surface plasmon excitation or the photonic superfocusing effect, which forms a nanosized spot of the light irradiation on plasmonic and photonic nanostructure surfaces. Using this small focusing spot as an optical source can allow imaging and manipulation with a subdiffraction-limit spatial resolution. However, the proposed near-field structures are constructed from high-index inorganic materials, such as noble metals and semiconductors^22,23^, which will mechanically damage the samples during near-field imaging and manipulation, especially biological cells and tissues. As an example, near-field optical scanning probes that are coated with a noble metal nanofilm or attached with a semiconductor nanowire or nanocavity will easily puncture the membranes of cells under near-field imaging and manipulation^22–24^. In addition, local heating induced by the optical absorption of the high-index materials will also cause undesired photothermal damage to biospecimens^25,26^. Moreover, few current near-field techniques can integrate both imaging and manipulation functions into a single device because of their complex nanostructures. Recently, a simple optical imaging scheme based on dielectric microspheres that can overcome the diffraction limit under conventional optical microscopes was widely investigated^27–34^. In 2011, Wang et al.^27^ demonstrated a 50-nm imaging resolution using microsphere nanoscopy for plasmonic samples with white-light illumination. The resolution of the microspheres was further improved to ~25 nm when coupled with a laser confocal microscope^28^. By using high-index immersed microspheres in aqueous environments, this technique has achieved rapid development in biological applications^29,30^, such as superresolution imaging of adenoviruses and subcellular structures. This microsphere-assisted imaging scheme is label-free; however, the current microspheres are commonly formed by artificially inorganic materials, such as silicon dioxide (SiO_2_), titanium dioxide (TiO_2_), and barium titanate (BaTiO_3_). A natural biomaterial is highly desired for the construction of a biocompatible and harmless device to achieve both imaging and manipulation with nanoscale spatial resolution.

Cells are naturally abundant biomaterials and are fully compatible with biological systems, which stimulated us to use living cells as optical devices in imaging and manipulation. Previous studies have reported that living cells can manipulate light in biological environments, acting as optofluidic microlenses^35,36^, optical probes^37,38^, and biophotonic waveguides^39,40^. However, the relatively small refractive index contrast and weak focusing ability of the above reported living cells prevent their application in nanoscale optical resolution. In this work, we show that the index contrast of living cells can be enhanced by having a spherical shape and being semi-immersed in a suspension to achieve subwavelength focusing ability. Combining the semi-immersed spherical cell with the interference enhancement effect by a mirror reflection, the focal spot size of the cell is extended beyond the half-wavelength diffraction barrier. Using this subdiffraction light spot to illuminate targeted samples, the near-field nanostructures of the samples could be magnified in the far field and captured by white-light microscopy. In this case, the living cell acts as a biomagnifier for nano-optical imaging. Furthermore, the nanosized light spot from the biomagnifier can exert a strong optical gradient force to trap and manipulate a single nanoparticle. Therefore, the biomagnifier can function as an optical nanotweezer.

## Results

### Schematic illustration and material characterization

**Fig. 1:**
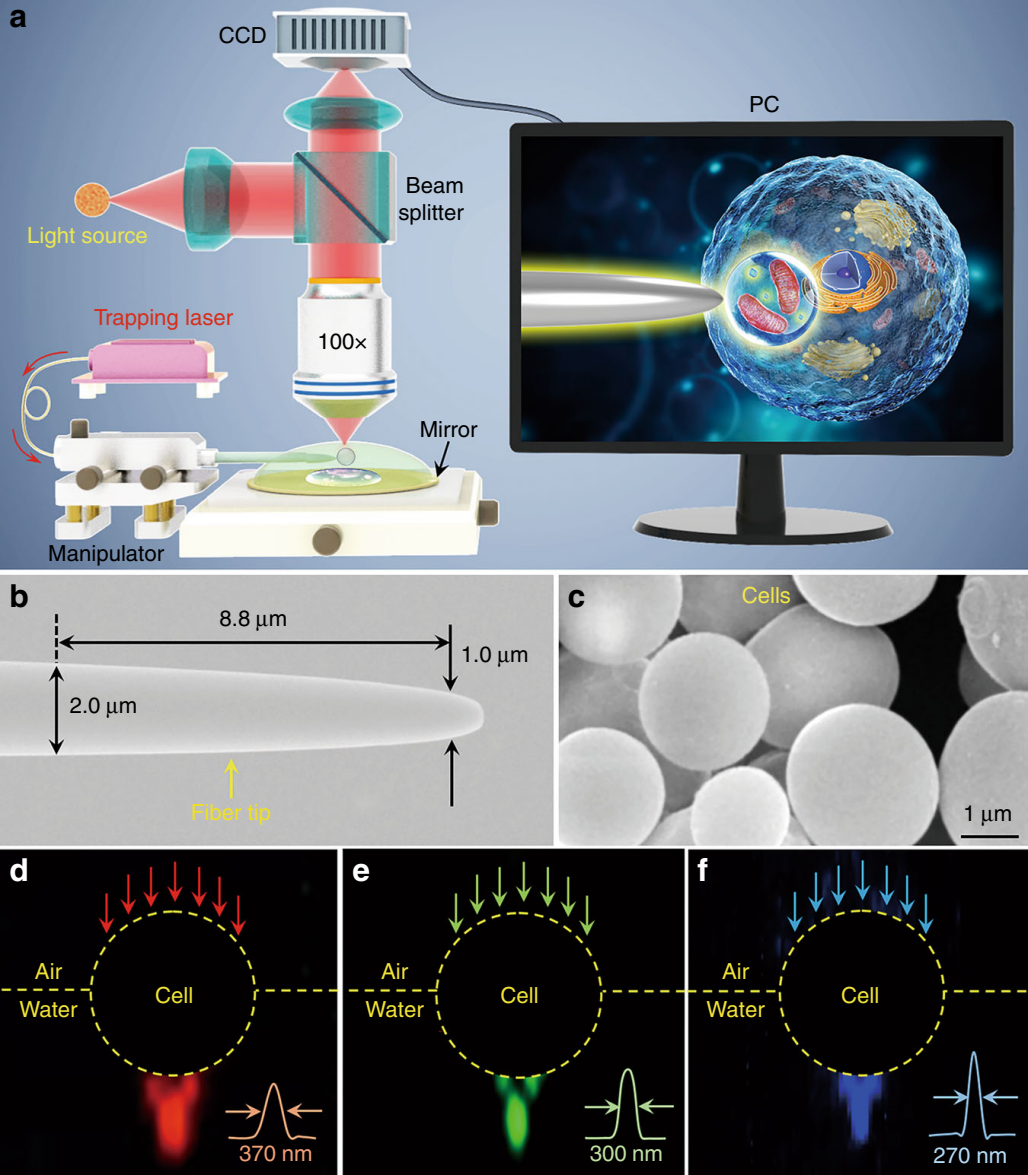
Schematic illustration and material characterization. **a** Schematic illustration of the experimental setup. A conventional reflection-mode microscope equipped with a CCD camera and ×100 objective lens was used to observe samples and record images. The inset shown in a PC screen schematically depicting how the biomagnifier is used to magnify and image the subcellular structures inside a biosample. **b** SEM image of the fiber tip with a diameter of 1.0 μm at its tapered end. **c** SEM image showing yeast cell-based biomagnifiers with smooth surfaces and spherical shapes. **d**-**f** Dark-field images showing 644-nm red light (**d**), 532-nm green light (**e**), and 473-nm blue light (**f**) transmitting through the biomagnifier and being focused into subwavelength light spots with waist radii of 370, 300, and 270 nm, respectively

Figure 1a presents a schematic of the experimental configuration. All the experiments were carried out under a reflection-mode optical microscope coupled with a charge-coupled device (CCD) camera and objective lens (NA = 0.95). An 390 nm ultraviolet (UV) light source, white-light halogen lamp at a center wavelength of 560 nm, and 808-nm semiconductor laser were used as the sources of excitation light, illumination light, and trapping light, respectively. The 808-nm laser was used for trapping because it exhibits relatively low absorption by biological specimens^3^. An optical fiber with a tapered tip (Fig. 1b) that was fabricated by drawing a commercial optical fiber was used to trap the biomagnifier at the end of the fiber (see Methods). The position of the trapped biomagnifier could be controlled by moving the fiber tip with a micromanipulator (highest accuracy: 50 nm per step). An inset on a personal computer (PC) screen schematically shows the imaging and manipulation of subcellular structures inside a biosample with the biomagnifier. To minimize the imaging aberration, the cells selected as biomagnifiers had smooth surfaces and spherical shapes, such as the yeast cells shown in Fig. 1c. To test the focusing capability of the biomagnifier, it was irradiated with visible light of different wavelengths. To directly visualize the output intensity distribution, monodisperse polystyrene (PS) nanoparticles were added to the cell suspension that could be illuminated by the output light from the biomagnifier (Fig. 1d–f). During this process, we observed an interesting phenomenon: the output light spot size gradually decreased when the cell was partially immersed in water. Supplementary experiments have been performed to investigate the effect of immersion depth on focusing of the cell. The immersion depth of the cell was determined with a monitoring measure. At the beginning, a certain volume of cell suspension was dropwise injected on the top of the sample through a micropipette until the cell was wholly immersed in the water droplet. As the water evaporated, the immersion depth of the cell gradually decreased. The evaporation process was monitored in real time using a lateral objective lens (magnification: ×60, NA: 0.73) and CCD camera. After the immersion depth reached the desired degree, the cell suspension was sealed by a polymer microchamber to prevent further evaporation. Supplementary Fig. S1 shows optical images of the cell with different immersion degrees. Although the cell exhibited better focusing performance when the immersion degree was lower than 1/2, a semi-immersed cell was selected in the imaging experiments because 1/2 immersion was more suitable for maintaining cell viability. Line intensity profiles along the focal spots (insets of Fig. 1d–f) revealed that the waist radii (*w*) of the spots from the semisubmerged biomagnifier were 370, 300, and 270 nm at the input wavelengths of 644, 532, and 473 nm, respectively, which indicated that the semisubmerged biomagnifier could focus light in the subwavelength region.

### Experimental imaging performance of the biomagnifiers

**Fig. 2:**
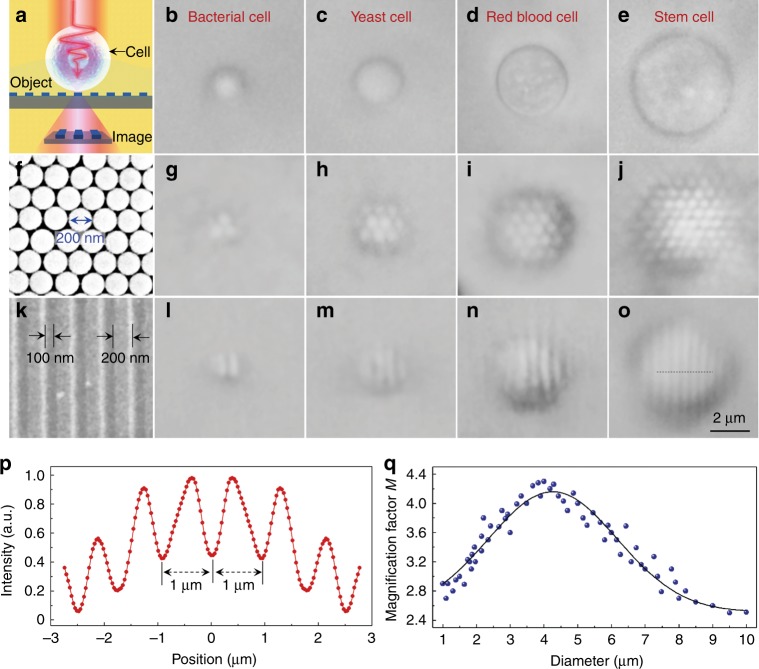
Experimental imaging performance of different biomagnifiers. **a** Schematic diagram showing that the biomagnifier collects the near-field nanostructures from an object and forms a virtually magnified image that can be captured by a conventional optical microscope. **b**–**e** Optical images of different biomagnifiers constructed from bacterial (**b**), yeast (**c**), red blood (**d**), and stem cells (**e**) that are partially submerged in cell suspension. **f** SEM image of a two-dimensional hexagonal close-packed silica nanosphere array assembled by a photophoresis technique. **g**–**j** Optical images of the silica nanosphere array magnified through biomagnifiers based on bacterial (**g**), yeast (**h**), red blood (**i**), and stem cells (**j**). **k** SEM image of the surface of a Blu-ray disk grating with a line width of 200 nm and spacing of 100 nm. **l**–**o** Optical images of the Blu-ray grating structure magnified through biomagnifiers based on bacterial (**l**), yeast (**m**), red blood (**n**), and stem cells (**o**). **p** Intensity profile along the dotted line across the Blu-ray grating structure indicated in **o**. **q** Blue dots showing the magnification factor *M* of the images obtained by the biomagnifiers as a function of the biomagnifier diameter

The experimental imaging setup of the biomagnifier is shown in Fig. 2a. A semisubmerged biomagnifier, positioned on the top of a test sample (a grating structure in this illustration), collects the underlying near-field information from the sample and then forms a virtual image, and it is detected by an optical microscope. To experimentally investigate their imaging performance as biomagnifiers, different species of cells, including bacterial, yeast, red blood, and stem cells (Fig. 2b–e), were prepared (see Methods). As the first imaging sample, a two-dimensional hexagonal close-packed silica nanosphere (diameter: 200 nm) array was assembled on a glass substrate using a photophoretic technique^41^ (Fig. 2f). As shown in Fig. 2g–j, only those nanospheres with biomagnifiers on top of them could be resolved. In contrast, the nanospheres without biomagnifiers could not be resolved by the conventional microscope owing to their size was smaller than the diffraction obstacle (for the peak illumination wavelength of 560 nm, the optical resolution is approximately 360 nm according to the Rayleigh criterion 0.61*λ*/NA). To enhance the optical resolution of the biomagnifier, we coated a gold film on the surface of the test sample because it could increase the light–matter interactions. For example, after coating with a gold film, the grating structure of a DVD disk (Fig. 2k) with 100-nm line spacing and 200-nm line width was resolved using the biomagnifiers (Fig. 2i–o). Compared with the size of the grating structure obtained from the scanning electronic microscope (SEM) image, the feature size imaged by the biomagnifier was clearly magnified with different magnification factors. Using a stem cell-based biomagnifier as an example, the intensity profile recorded by the biomagnifier along the dotted line in Fig. 2o showing the grating period of the DVD disk was 1.0 μm (Fig. 2p), which was 3.3 times larger than that obtained by the SEM image (grating period: 300 nm). Thus, the magnification factor *M* of the stem cell-based biomagnifier was determined to be ×3.3. The experimental *M* depended on the diameter of the biomagnifier. As shown in Fig. 2q, the largest *M* (approximately ×4.0) was obtained when the biomagnifier diameter was ~4 μm. Therefore, the subsequent experiments were performed using biomagnifiers with this diameter.

**Fig. 3:**
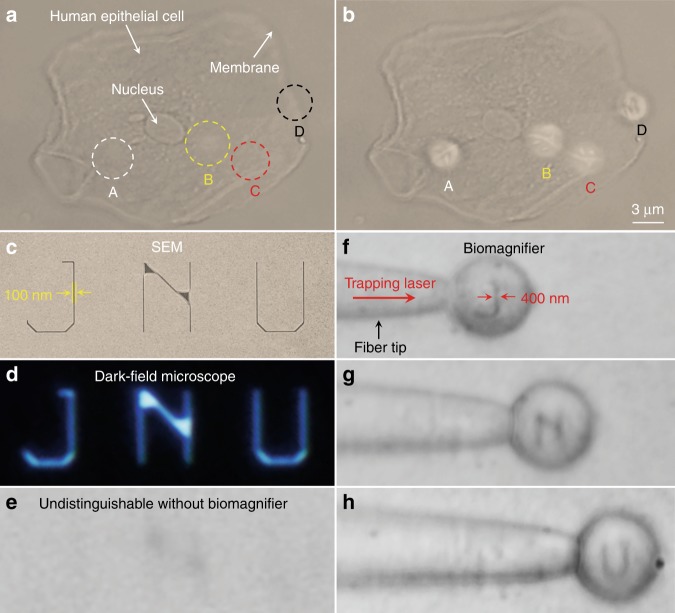
Nano-optical imaging of subcellular structures and nanopatterned letters. **a**, **b** Optical images of the subcellular structures of a human epithelial cell using a conventional optical microscope (**a**) and biomagnifiers (**b**). The positions of four biomagnifiers are marked as A–D. For comparison, the biomagnifiers can resolve the fibrous cytoskeleton (indicated as A–C) inside the cell and two-layer structures (indicated as D) on the cell membrane, which are indistinguishable by the conventional microscope. **c**–**e** SEM (**c**), dark-field (**d**), and optical images (**e**) of nanopatterned letters JNU, which represent the acronym of Jinan University. The line width of the nanopatterned letters is 100 nm, which is smaller than the diffraction-limit resolution of the conventional optical microscope. **f**–**h** Optical images showing that the biomagnifier trapped on the fiber tip can scan and image the nanopatterned letters J (**f**), N (**g**), and U (**h**) by moving the fiber. The line width of the nanopatterned letters was magnified from 100 to 400 nm

To investigate the applicability of biomagnifiers to biological imaging, we performed an imaging experiment using human epithelial cells as imaging targets. The epithelial cells were grown on a mirror substrate to enhance the light–matter interaction by the interference effect of the illumination light and reflection light. Under a conventional optical microscope, it was difficult to distinguish the fibrous cytoskeleton inside the cell (indicated as A–C in Fig. 3a) and the bilayer structures on the cell membrane (indicated as D in Fig. 3a). After positioning biomagnifiers on the top of the epithelial cell, the fibrous cytoskeleton (indicated as A–C in Fig. 3b) and bilayer membrane (indicated as D in Fig. 3b) of the cell were resolved. Although the optical imaging of these subcellular structures has been previously achieved by superresolution fluorescence microscopes, the biomagnifier provides a direct imaging approach without labeling the cells with specific fluorescent molecules. The imaging field of view (FOV), which was defined as the region that could be observed by the biomagnifier, was limited to the size of the biomagnifier. To improve the FOV, the biomagnifier was trapped on a fiber tip and then was moved to scan the samples. Here, we used the nanopatterned letters JNU (an acronym of Jinan University) as the test sample for scanning imaging; the letters were prepared on a silicon substrate using electron-beam lithography. An SEM image showed that the line width of the nanopatterned letters was 100 nm and that the effective area of these letters was approximately 100 μm^2^ (Fig. 3c). Although the nanopatterned letters could be observed by a dark-field scattering microscope (Fig. 3d), because of the strong scattering of silicon material, they could not be discerned by a conventional optical microscope, especially when they were immersed in water (Fig. 3e). By manipulating the trapped biomagnifier to scan the nanopatterned letters at a rate of ~20 μm/s, each of the letters was able to be resolved within 2 s (Fig. 3f–h). With assistance of the biomagnifier, the line width of the nanoletters was magnified from 100 to 400 nm; i.e., *M* was ×4.

Using this scanning strategy, the biomagnifier is expected to allow wide-field imaging by reconstructing the images produced by each scanning result to form a complete image. Compared with point-based scanning imaging methods (e.g., scanning near-field optical microscopy), the biomagnifier uses an “area” instead of the “point”, which can markedly improve the time efficiency. Interestingly, because of the intrinsic elasticity of the cells, the morphology of the biomagnifier could be changed by raising the optical gradient force by increasing the trapping power. Changing the shape of the biomagnifier allowed its focal length to be adjusted from 0.7 to 5.0 μm (see Supplementary Fig. S2). This tunable focusing ability of the cell-based biomagnifier is expected to allow the two-dimensional imaging (*x–y* plane) to be extended to three-dimensional (3D) imaging by adding axial scanning in the *z* direction. Additional experiments were conducted to assess the reproducibility of this imaging technique (see details in Supplementary Fig. S3). In the repeated tests, red blood cells were trapped by optical tweezers and then manipulated to repeatedly image the same target sample (i.e., Blu-ray grating structure). The repeat times were determined once the grating structure could not be resolved by the cells. The results show that the trapped cell could be utilized for imaging for over 300 times at an optical power of 10 mW. The repeat times could further increase with lower optical powers. In the experiments, the illumination angle *θ* was calculated as 71.8° according to NA = *n* sin (*θ*/2), where NA is equal to 0.95 and *n* denotes the refractive index. For comparison, a larger *θ* (i.e., 90°) was incident by an objective with NA of 1.0. The result shows that a larger illumination angle could improve the imaging quality (see details in Supplementary Fig. S4), because the objective lens with larger illumination angle could excite and collect higher spatial frequencies containing detailed information of nanostructures.

### Optical manipulation of single nanoparticles

**Fig. 4:**
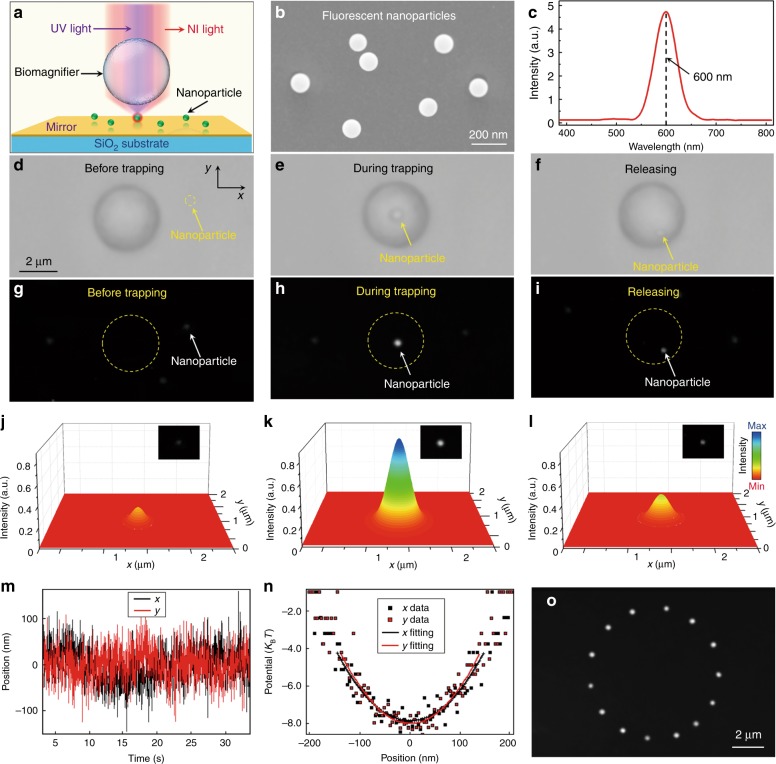
Optical manipulation of a single fluorescent nanoparticle. **a** Schematic diagram showing a fluorescent nanoparticle suspended on the surface of a mirror and trapped by the biomagnifier. **b** SEM image showing the PS fluorescent nanoparticles with an average radius of 50 nm. **c** Emission spectrum showing the central emission wavelength of the fluorescent nanoparticles located at 600 nm. d–f Optical images show the trapping process of a single PS nanoparticle with the biomagnifier. The process consisted of three successive steps: before trapping (**d**), during trapping (**e**), and after release (**f**). **g**–**i** Fluorescence images showing the fluorescence spot of the PS nanoparticle before being trapped (**g**), during trapping (**h**), and after release (**i**). **j**–**l** Three-dimensional color mapping of the fluorescence spots of the nanoparticle as shown in **g**–**i**. **m** Real-time trace of the position of the trapped nanoparticle in the *x* and *y* directions. **n** Trapping potential of the trapped nanoparticle in the *x* and *y* directions with parabola fittings. **o** Composite fluorescence images show the movement trace of the trapped nanoparticle in the *x–y* plane by controlled movement of the biomagnifier

The subwavelength light spot focused by a biomagnifier also allows us to trap and manipulate nanoparticles in three dimensions. Figure 4a shows the optical setup for trapping and manipulation experiments. Near-infrared and UV laser beams were simultaneously irradiated on the biomagnifier through a microscope objective lens to trap and excite the nanoparticles, respectively. The measured input power was 10 mW at the focal region of the microscope. The spot size focused by the microscope objective was approximately 2 μm, and thus, the estimated power density was 1 × 10^10^ W/m^2^, which was much smaller than that of standard optical tweezers (generally from 1 × 10^11^ to 1 × 10^12^ W/m^2^)^17^. To directly observe the nanoparticles under the optical microscope, fluorescent PS nanoparticles were selected as the test samples (see Methods for preparation details), which were suspended on a mirror substrate. The average radius (*a*) of the fluorescent PS nanoparticles from measurements was 50 nm (Fig. 4b), which was in the Rayleigh regime (*ka* *=* 2π*a*/*λ* < 0.8)^42^. When excited by the 390-nm UV light, the nanoparticles launched fluorescence light at 600 nm wavelength (Fig. 4c). Bright-field optical images (Fig. 4d–f) and dark-field fluorescence images (Fig. 4g–i) show the trapping process of a single fluorescent PS nanoparticle by the biomagnifier. Before trapping, the nanoparticle could not be directly detected under the microscope due to the diffraction limit of light (Fig. 4d), although a weak fluorescence spot from the nanoparticle could be seen in fluorescence mode (Fig. 4g). When a single nanoparticle was trapped in the focus of the biomagnifier, the nanoparticle was able to be clearly seen in both optical (Fig. 4e) and fluorescence (Fig. 4h) images because of the magnification mechanism of the biomagnifier. After being released, the nanoparticle moved from the focus of the biomagnifier because of its intense Brownian motion in the water environment (Fig. 4f). In this case, the size of the nanoparticle and intensity of fluorescence observed under the microscope decreased compared with the case for the trapped nanoparticle (Fig. 4i). Figure 4j–l shows the 3D color mapping of nanoparticle fluorescence before being trapped, during trapping, and after release, respectively. The total optical intensity of the fluorescence spots was calculated by performing a surface integral of the color mapping. The total fluorescence intensity of the trapped nanoparticle was enhanced with enhancement factors of 70 and 30 when compared with that of the nanoparticle before being trapped and after release, respectively. This fluorescence enhancement benefited from the focusing ability of the biomagnifier, which increased the excitation intensity of the UV light and improved the collection efficiency of the fluorescence signal.

To calculate the trapping stiffness *κ*_trap_ of the particle trapped by the biomagnifier, the location of the nanoparticle during trapping was recorded in real-time by a four-quadrant photodiode in a standard optical tweezers system (Aresis Tweez 250si). The position vibration of the trapped nanoparticle was induced by its inherent Brownian motion (Fig. 4m). According to the energy equipartition theorem^43^, *κ*_trap_ can be obtained from the position variance 〈*σ*^2^〉 of the Brownian motion:

1 $$\frac{1}{2}k_{\mathrm{B}}T = \frac{1}{2}\kappa _{{\mathrm{trap}}}\left\langle {\sigma ^2} \right\rangle$$

where *k*_B_ denotes Boltzmann’s constant and *T* indicates the normal temperature. 〈*σ*^2^〉 was determined by the quadratic coefficient of parabola fitting to the trapping potential versus position (Fig. 4n). The calculated 〈*σ*^2^〉 values of the nanoparticle in the *x* and *y* directions were 98 and 95 nm, respectively, which indicated that the position precision of the trapped nanoparticle was below 100 nm. Substituting these 〈*σ*^2^〉 values into Eq. (1) gave experimental *κ*_trap_ in the *x* and *y* directions of the nanoparticle of 0.41 and 0.42 pN/nm/W, respectively. *κ*_trap_ obtained by the biomagnifier is twice that determined using slot waveguides (*κ*_trap_ = 0.21 pN/nm/W)^44^ and much larger than those of traditional optical tweezers (*κ*_trap_ = 0.0014 pN/nm/W)^43^ and plasmonic tweezers (*κ*_trap_ = 0.0012 pN/nm/W)^45^. Here, the *κ*_trap_ values were scaled to a nanoparticle with a radius of 50 nm because the trapping force is in direct proportion to the third power of the particle size^46^. The trapped nanoparticle could be three-dimensionally manipulated by adjusting the biomagnifier (see motion trajectory in Fig. 4o and Supplementary Movie S1). This contactless and precise manipulation of a single nanoparticle will be useful in optical assembly of well-regulated nanostructures.

### Numerical analysis of the imaging mechanism and trapping stiffness

**Fig. 5:**
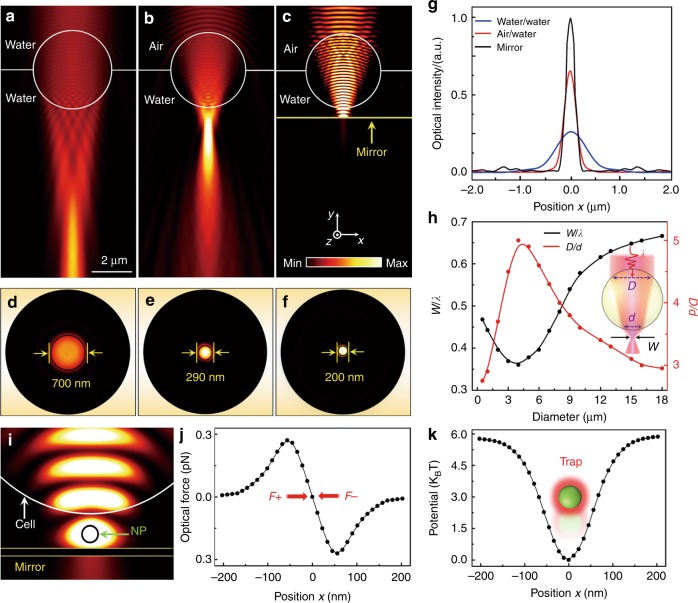
Numerical simulation and calculation. **a**–**c** Optical intensity distributions of light focusing by a 4-μm biomagnifier fully immersed in water (**a**), semi-immersed in water (**b**), and suspended on the surface of a mirror (**c**). The illumination light source was set as a Gaussian beam with a wavelength of 560 nm. **d**–**f** Optical intensity distributions of the light spots from the biomagnifier corresponding to **a**–**c**) in the *x–z* plane. **g** Optical intensity profiles at the focal planes of the output light from the biomagnifiers in the *x* direction. **h** FEM simulation results for the normalized waist of the light spot *w*/*λ* (*w* is the waist radius of the light spot and *λ* is the wavelength of the input light) and the ratio *D*/*d* (the width of the linear region where light enters the biomagnifier at its front surface is referred to as *D*, and the width of the output light beam at the rear surface is *d*) as a function of the biomagnifier diameter. **i** Simulated intensity distribution of near-infrared trapping light showing that a nanoparticle (radius: 50 nm) is trapped in the gap between the biomagnifier and mirror. The input optical power of the trapping light was set to 10 mW. **j** Simulated optical forces of the nanoparticle trapped in the light spot as a function of the nanoparticle position along the *x* direction. **k** Calculated trapping potential of the trapped nanoparticle as a function of the position along the *x* direction

The imaging mechanism and trapping stiffness of biomagnifiers were numerically investigated by performing a 3D simulation and calculation using COMSOL software (see Methods). The optical intensity (*I*) distribution of the illumination light (*λ* = 560 nm) irradiated onto a fully immersed biomagnifier shows that the light was focused in the far field with a focal length *L* of 7.0 μm (*L* > 10*λ*), resulting in a relatively large output light spot (Fig. 5a). When the middle of the biomagnifier was placed at the air–water interface, the output light was highly focused in the near field with a focal length of 0.7 μm (*L*_*;*_*≈λ*) and formed a tiny light spot (Fig. 5b). Furthermore, by positioning a mirror substrate under the biomagnifier with a gap of 300 nm (corresponding to the experimental parameters), the optical intensity of the light spot further increased because of the interference enhancement between the input light of the biomagnifier and reflection light of the mirror (Fig. 5c). *I* distributions of the output light spots in the focal plane (*x–z* plane) revealed that the *w* of the spots were 700, 290, and 200 nm for the fully immersed biomagnifier (Fig. 5d), bare semi-immersed biomagnifier (Fig. 5e), and semi-immersed biomagnifier above the mirror (Fig. 5f), respectively. In particular, the *w* of the output light spot from the semi-immersed biomagnifier with the mirror overcame the half-wavelength diffraction limit of the illumination light. This subdiffraction-limit light focusing ability resulted from the combination of the “photonic nanojet” effect^47–49^ of the spherical biomagnifier and the coherent interference enhancement by the mirror. Yang et al.^31^ demonstrated that *w* of a photonic nanojet governs the imaging resolution *R* by

2 $$R = \frac{w}{{2\sqrt {2\ln \left( 2 \right)} }}$$

For the photonic nanojet focused by the semi-immersed biomagnifier above the mirror (*w* = 200 nm), *R* was ~85 nm, which was slightly smaller than the experimental resolution (100 nm). This deviation of the imaging resolution is mainly because the geometric configuration and refractive index of the biomagnifier were respectively defined as perfectly symmetrical and homogeneous in the simulations. We also performed simulations to investigate the relationship between the focusing capability and magnification factor (*M*) of the biomagnifiers. Figure 5h shows that the focusing ability of the biomagnifiers was defined by the waist radius of the output light spot normalized with the input wavelength (*w*/*λ*), and the *M* was indicated by the ratio *D*/*d*, where *D* denotes the distance of the linear region where beam entered the biomagnifier, and *d* denotes the distance of the output light at the shadow surface of the biomagnifier (inset of Fig. 5h). According to the variation tendency of the profiles, the biomagnifier with a radius of ~2 μm showed the optimal focusing ability and minimum size of the output light spot, consistent with the experimental result whereby the biomagnifier with a radius of ~2 μm had the largest *M*.

To numerically investigate *κ*_trap_ of a nanoparticle trapped by a biomagnifier, we placed a 50 nm PS particle in the focusing spot of a biomagnifier above a mirror (Fig. 5i). The light spot was one of the interference fringes from the input light and reflection light, exerting an optical gradient force (**F**_o_) on the nanoparticle, which can be expressed as^50^

3 $${\mathbf{F}}_{\mathrm{o}} = {\oint}_S {\left( {\left\langle {{\mathbf{T}}_{\mathrm{M}}} \right\rangle \cdot {\mathbf{n}}} \right){\mathrm{d}}{\it{S}}}$$

where the closed integration is carried out over a surface *S* surrounding the particle; **n** denotes the normal unit vector; 〈**T**_M_〉 is the Maxwell stress tensor:

4 $$\left\langle {{\mathbf{T}}_{\mathrm{M}}} \right\rangle = \frac{1}{2}{{\mathrm{Re}}} \left[ {\varepsilon {\mathbf{EE}}^ \ast + \mu {\mathbf{HH}}^ \ast - \frac{1}{2}\left( {\varepsilon \left| {\mathbf{E}} \right|^2 + \mu \left| {\mathbf{H}} \right|^2} \right)q} \right]$$

where HH* and EE* indicate the outer product of the electromagnetic fields; *ε* is the electric permittivity; *μ* is the magnetic permeability; *q* denotes the unit dyadic. Figure 5j shows the simulated **F**_o_ *versus* the position *x*. By measuring the linear slope at *x* = 0 from the optical force profile, the simulated *κ*_trap_ was estimated to be 0.46 pN/nm/W in the *x* direction, which agrees with the experimental *κ*_trap_ of 0.41 pN/nm/W. To demonstrate the trapping stability, optical potential *U*_*x*_ was evaluated by integrating *F*_o_ over the position *x* of the nanoparticle according to

5 $$U_x = - {\int} {F_{\mathrm{o}}{\mathrm{d}}x}$$

The calculated *U*_*x*_ in the *x* direction is presented in Fig. 5k with a potential depth (Δ*U*) of approximately 6.0 *k*_B_*T*. After the nanoparticle drops into this trap, it will be confined until the thermal potential surpasses the largest trapping potential depth. Ashkin et al.^51^ demonstrated that a stable trap needs a Δ*U* of 1–10 *k*_B_*T*. Therefore, the potential depth of the biomagnifier (Δ*U* = 6.0 *k*_B_*T*) was suitable to form a stable trap.

## Discussion

The experimental imaging resolution of this technique was determined to be ~*λ*/5.5 using a convolution process, as described by Allen et al.^52,53^ and Darafsheh et al.^54,55^. Here, a gold dimer containing two 100-nm particles with an edge-to-edge distance of 50 nm was selected as the test sample, which was prepared on a sapphire substrate. An optical image of the dimer was obtained by placing a 4-μm red blood cell on the top of the sample. The optical image could be considered as the convolution between the point-spread function (PSF) of the optical system and the intensity distribution function of the samples. Using a Gaussian function for the PSF to fit the intensity distribution of the gold dimer, the imaging resolution was determined as the waist radius of the PSF, which is on the order of λ/5.5 (see details in Supplementary Fig. S5). To further investigate the imaging ability, we also carried out an FEM simulation, which was basically modeled by placing two point sources between the gap of the sample and substrate (see details in Supplementary Fig. S6), as described by Maslov and Astratov^56,57^. The imaging process was described as that the near-field evanescence wave of nanostructures (equivalent to the point sources) converted into a propagating wave through the cell-based lens, which was then collected by a microscope objective, forming a virtual image on a CCD. The imaging resolution limit was calculated as the smallest distance between the point sources, which could be resolved by Houston criterion. As a result, the theoretical resolution limit for the cell-based lens was approximately λ/4.5. The theoretical resolution limit was smaller than the experimental resolution obtained by the convolution process. We assumed that this is because the interference effect of the mirror substrate or gold nanofilm used in the imaging experiments could help improve the imaging resolution. Because of the interference enhancement of the input light and reflection light, *w* of the focal light spot was compressed with a factor of 1.5. A smaller focal light spot would have resulted in a better imaging resolution. Moreover, the illumination light was transmitted through the samples twice owing to the reflection, which could enhance the light–matter interactions, also enabling a better imaging quality. This interference enhancement mechanism was also verified by Yang et al.,^58^ who exploited a mirror to enhance the axial and lateral imaging resolution of biosamples under stimulated emission depletion nanoscopy.

The viability of the cells was an important issue for this technique. There are some differences in using a live cell and a dead cell in our experiments. For the live cell, it could maintain a sphere in liquid environments because of its inherent membrane elasticity, which was crucial for the cell to function as an imaging lens. For the dead cell, the cell membrane would gradually become less elastic, and the intracellular materials would diffuse outside from the cell. Therefore, the morphology and refractive index of the dead cell would finally become irregular and inhomogeneous, influencing the imaging resolution. To maintain the cell viability, a moderate optical power that the cells could endure was applied for trapping and imaging. Additional experiments have been performed to measure the temperature rise inside the cell by using upconversion fluorescence nanoparticles (UCNPs) (see details in Supplementary Fig. S7). The UCNPs (NaYF_4_:Yb^3+^/Tm^3+^) with an average radius of 10 nm were used to label the cells through surface modification and endocytosis effect, as described in our previous work^36^. Because the upconversion fluorescence was sensitive to the temperature of the medium, the UCNPs were widely used for temperature detection. The experimental results show that for the 10 mW optical power, the temperature increment of the cells after irradiation for 2 h was measured as approximately 1.3 °C. Such a low temperature increase would not damage the cells during the trapping and imaging. Moreover, the cell viability could be monitored with trypan blue staining assays (Supplementary Fig. S8). Dead cells would absorb trypan blue and become distinguishable from live cells. The results indicate that the cells under irradiation with an optical power that was smaller than 45 mW for 2 h were still alive after the experiments. However, when irradiated with an optical power that was larger than 45 mW for 2 h, the cells were dead and stained blue. Therefore, the operational power threshold in our technique was 45 mW, which could serve as a reference for further investigation.

Compared with our previous publications, this work has achieved some substantial progress. (1) This work integrates optical nanoscopes and nanotweezers into a single device, allowing us to simultaneously image and manipulate nanostructures, which was never achieved by the previous works. (2) The imaging resolution of this technique was promoted to 100 nm, which was nearly two times higher than that of our previous technique (~190 nm)^38^. (3) This work proposed a label-free imaging technique, while in our previous works, the biosamples were labeled with fluorescence markers, such as UCNPs or green fluorescence protein^36,38^. To evaluate this technique more comprehensively, here, we discuss the limitations of the cell-based lens and propose some possible solutions. First, when compared with dielectric microspheres having a fully uniform refractive index, the inhomogeneous structures inside the cells could cause a certain degree of imaging aberration and distortion because the inhomogeneous refractive index prevents perfect light focusing. Fortunately, however, most of the materials inside the cells are optically transparent for visible and near-infrared light; thus, the optical interactions, including absorption and scattering by light, are relatively weak inside a single cell. Especially for bacterial cells and red blood cells, the intracellular refractive index was relatively homogeneous because these cells lack a nucleus and organelles. Second, due to the inherent membrane elasticity, the shapes of the cells could be tuned by light with a relatively high optical power. This effect could be avoided by using a moderate optical power such as the 10 mW power used in our imaging experiments. From another point of view, the change of the cell shape could pave the way for assembling a tunable lens, which was a unique property of the cell-based lenses and could not be achieved by the inorganic microsphere lenses. Third, some specific types of activities inside the cell would influence the trapping and imaging performance. For example, the cell activities of organelle movement and cell endocytosis would change the partial refractive index distribution inside the cell and thus cause light distortion during trapping and imaging. However, this influence was not obvious because most of the organelles and endocytosis contents were relatively small when compared to the illumination wavelength. Some other cell activities, such as cellular respiration, protein transport, or DNA replication, were ultrafast processes and could not be observed under the optical microscope; thus, these activities had no influence on the trapping and imaging scheme.

In conclusion, we demonstrated that spherical semi-immersed cells trapped on a fiber tip can function as natural biomagnifiers for nano-optical imaging and manipulation of nanostructures. Through illumination by the subdiffraction-limit light spot of the biomagnifier, nanostructures on a mirror can be imaged in real time with an experimental resolution of 100 nm. Moreover, the biomagnifier could be applied for stably trapping and manipulation of a 50-nm fluorescent particle. This living biomagnifier is envisioned to open opportunities in superresolution imaging, real-time sensing, and precise assembly of bionanomaterials, such as small pathogenic bacteria, viruses, and biomolecules.

## Materials and methods

### Preparation of the optical fiber tip

The silica fiber tip was prepared using a one-step drawing method^59^. Before being heated, a bare optical fiber with a length of 2.5 cm was obtained by stripping off the outer covering layer of the optical fiber. To protect the bare optical fiber, it was sheathed with a quartz microtube. The bare fiber was heated using an alcohol lamp for approximately 30 s. The bare fiber was then pulled with a velocity of ~4 mm/s, which caused it to gradually taper off (the diameter of the tapered region was ~10 μm). The pulling velocity was improved to ~ 20 mm/s within 0.1 s, and then the bare fiber broke with a tapered microtip.

### Cell culture

Bacteria and yeast (Shanghai Ruichu Biotech Co., Ltd.) were cultured at 37 °C overnight in lysogeny-broth suspension. Then, the bacteria and yeast suspension were washed for three times and diluted to a concentration of approximately 5.0 × 10^4^ cells/μL. The red blood cells were separated by centrifugation at 3500 r/min for 15 min from human whole blood, which was obtained from an adult volunteer and diluted using normal phosphate-buffered saline solution with a pH value of 7.45. The stem cells and epithelial cells were grown on Petri dishes in Dulbecco’s modified Eagle's medium with a mixed solution of fetal bovine serum (10%) and penicillin–streptomycin (1%) at 37 °C with a CO_2_ concentration of 5%.

### Preparation of the fluorescent nanoparticles

The particles (purchased from Shanghai Huge Biotechnology Co., Ltd.) dyed with organic fluorescence molecules had a peak emission wavelength of 600 nm with 390-nm excitation. Then, the fluorescent particles were diluted to a concentration of approximately 5.5 × 10^4^ particles/μL. The prepared particle solution was injected onto a mirror substrate using a micropipette (accuracy: 0.1 μL) for the trapping experiments.

### Simulated analysis

The simulations were performed using a 3D FEM in COMSOL software with the radio frequency-domain module and perfectly matched layer boundary. The input light was set as an unpolarized Gaussian beam. The biomagnifier used in the simulations was assumed to be a sphere (diameter: 4.0 μm). The mesh sizes of water, biomagnifier, PS particle, and mirror were 80, 50, 10, and 10 nm, respectively, and the corresponding refractive indices were 1.33, 1.40 (ref. ^35^), 1.58, and 0.34 + 2.75*i* for the wavelength of 560 nm. For the wavelength of 808 nm, the refractive index of the mirror was 0.16 + 5.18*i* (ref. ^60^).

## Supplementary information

Supplementary Materials.

Supplementary Movie S1.
