# Supplementary material for: Single-cell biomagnifier for optical nanoscopes and nanotweezers
Source: Light Sci Appl. 2019 Jul 3;8:61. doi: 10.1038/s41377-019-0168-4 (PMC6804537; doi:10.1038/s41377-019-0168-4)
Supplement: Supplementary file 1 — Supplementary Materials. [file 41377_2019_168_MOESM1_ESM.docx]

Supplementary Materials

**Single-cell biomagnifier for optical nanoscopes and nanotweezers**

Yuchao Li, Xiaoshuai Liu & Baojun Li*

Institute of Nanophotonics, Jinan University, Guangzhou 511443, China.

*Email: [baojunli@jnu.edu.cn](mailto:baojunli@jnu.edu.cn)

**1. Cell-lens immersion issue and focusing performance.**

Additional experiments have been performed to investigate the effect of immersion depth on focusing of the cell. As shown in Fig. S1a–d, the waist radii of the focal light spots from the cell with 1/4, 1/3, and 3/4 immersion were measured as 330, 355, and 480 nm, respectively, at the illumination wavelength of 644 nm. The immersion depth of the cell was determined with a monitoring measure. At the beginning, a certain volume of cell suspension was dropwise injected on the top of the sample through a micropipette until the cell was wholly immersed in the water droplet. As the water evaporated, the immersion depth of the cell gradually reduced. The evaporation process was monitored in real time by using a lateral objective lens (magnification: ×60, NA: 0.73) and CCD camera. After the immersion depth reached the desired degree, the cell suspension was sealed by a polymer microchamber to prevent the further evaporation. Figures S1e–h present the optical images of the cell-based lens with 3/4, 1/2, 1/3 and 1/4 immersion degree. Figure S1i shows the waist radius of the focal light spot versus the cell immersion degree.


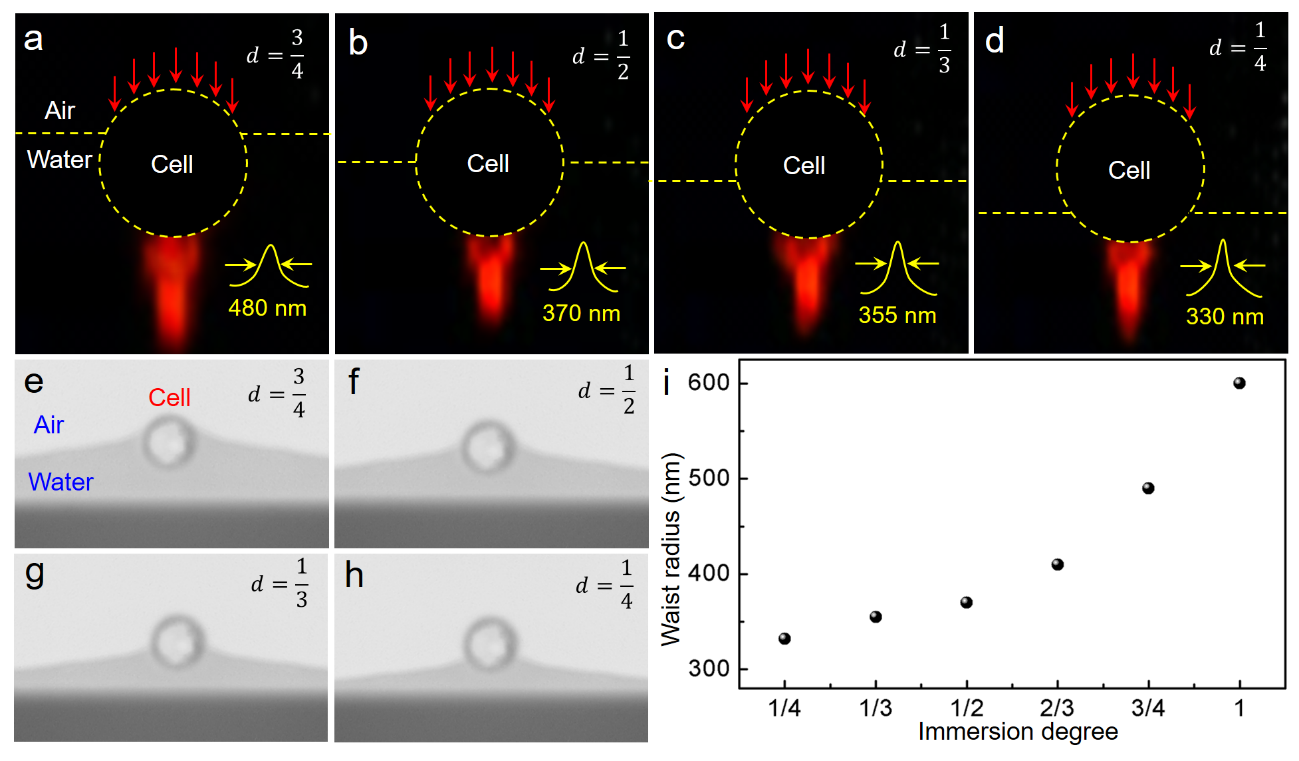


**Figure S1 | Immersion issue and focusing performance.** (**a**–**d**) Optical dark-field images showing 644-nm red light transmitting through a cell-based lens and being focused into light spots with waist radii of 480, 370, 355, and 330 nm under the immersion degree of 3/4 (a), 1/2 (b), 1/3 (c), and 1/4 (d), respectively. (**e**–**h**) Optical images of the cell-based lens with 3/4 (e), 1/2 (f), 1/3 (g), and 1/4 (h) immersion degree. (**i**) Waist radius of the focal light spot as a function of the cell immersion degree.

**2. Focal length tunability of the biomagnifier by adjusting the trapping powers.**

When the biomagnifier was trapped to the fiber tip, an optical trapping force will press the biomagnifier against the fiber. The optical force became stronger by increasing the trapping powers. Due to the intrinsic deformable property of the cell-based biomagnifier, the shape of the biomagnifier could be changed from a sphere to an ellipsoid. For example, the deformation factor *D*, which was defined as the ratio of the long axis *b* and short axis *a* of the biomagnifier, could be changed from 1.2 to 1.7 by increasing the trapping power from 50 to 80 mW (Fig. S2a & b). Numerical simulation demonstrated that the focal length of the biomagnifier could vary with the change of the deformation factor (Fig. S2c & d). More specifically, the deformation factor *D* could be increased from 1.0 to 1.8 with trapping powers increasing from 20 to 180 mW (Fig. S2e), which results in focal length changing from 0.7 to 5.0 μm (Fig. S2f).


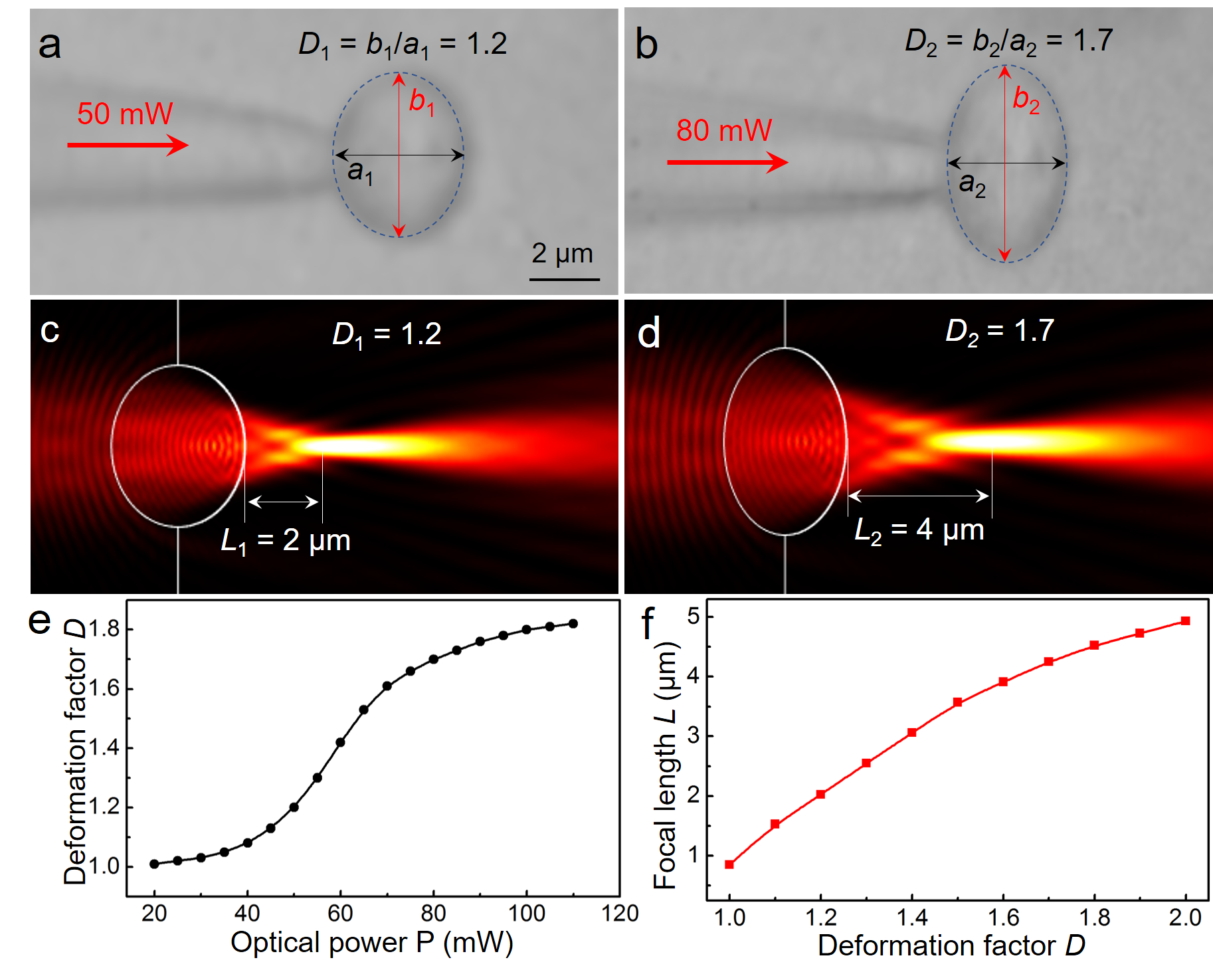


**Figure S2 | Tunable focal length of the biomagnifier.** (**a**,**b**) Optical images showing the shape of the biomagnifier was changed by increasing the trapping power from 50 to 80 mW. (**c,d**) Simulated intensity distributions of outputted light from the biomagnifier, which correspond to the images in (a) and (b). (**e**) Deformation factor (*D*) of the biomagnifier as a function of the trapping power (*P*). (**f**) Focal length (*L*) of the biomagnifier as a function of *D*.

**3. Reproducibility tests of the imaging technique.**

In the repeated tests, red blood cells were trapped by optical tweezers and then manipulated to repeatedly image the same target sample (*i.e.*, Blu-ray grating structure). The time interval between two imaging process was set as 30 s, and the repeat times was determined once the grating structure could not be resolved by the cells. Figures S3a–c show the optical images of the grating structure obtained by the cell at the 1st, 150th, and 300th time with an optical power of 10 mW. Figure S3d shows the repeat times could a cell be utilised for imaging at different optical power levels. At each optical power, three cells were used to image the same sample, and then the mean and variance of the repeat times were obtained. The results show that the repeat times could further increase with lower optical powers.


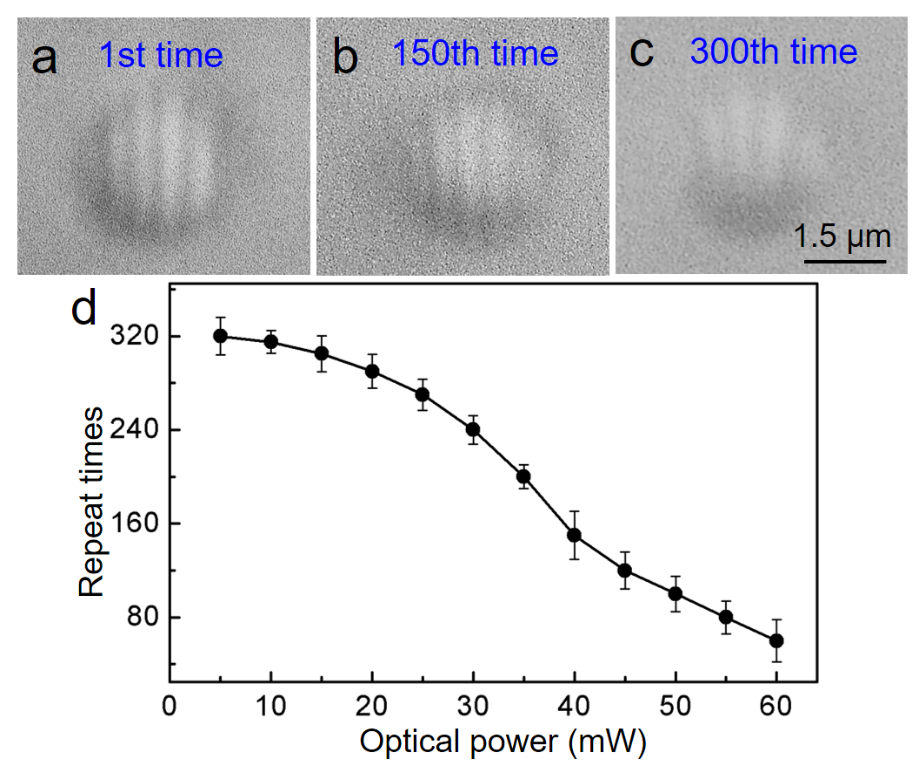


**Figure S3 | Reproducibility tests.** (**a**–**c**) Optical images of the grating structure obtained by the red blood cell at the 1st, 150th, and 300th time with an optical power of 10 mW. (**d**) Repeat times as a function of the optical power.

**4. The influence of numerical aperture and illumination angle on optical imaging.**

In the experiments, the illumination angle *θ* was calculated as 71.8º according to NA = *n*·sin (*θ*/2), where *n* is the refractive index and NA is equal to 0.95*.* For comparison, a larger *θ* (*i.e.*, 90º) was incident through an objective lens with NA of 1.0. The result shows that larger illumination angle could improve the imaging quality, as shown in Fig. S4a and b.


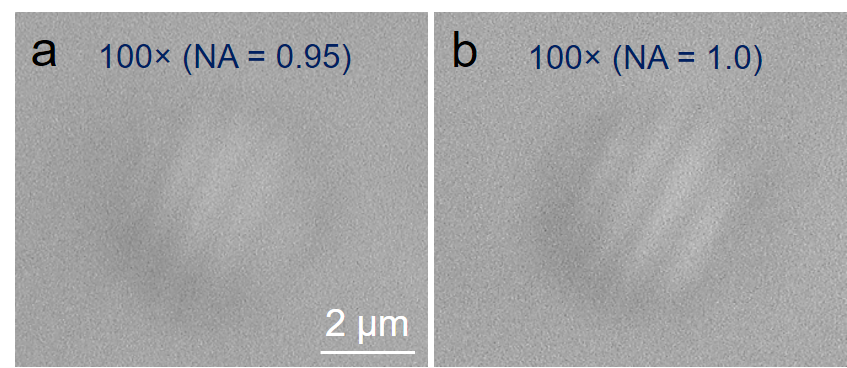


**Figure S4 | Optical imaging with objective lenses with different numerical apertures.** Optical images of grating structures obtained by a cell-based les under an objective lens with numerical aperture of 0.95 (**a**) and 1.0 (**b**).

**5. Experimental imaging resolution determined by using a convolution process.**

The experimental imaging resolution of this technique could be determined by using a convolution process. As shown in Fig. S5a, a gold dimer containing two 100-nm nanocylinders with 50 nm edge-to-edge separation was selected as the test sample, which was fabricated on a sapphire substrate by an electron beam lithography and metal evaporation. An optical image of the dimer was obtained by placing a 4-μm red blood cell on the top of the sample (Fig. S5b). Intensity profile along the *x*-axis of the dimer was illustrated using red as color in Fig. S5c. The saddle-to-peak ratio of the intensity profile was measured as 0.35, which was smaller than that assumed in classical definition of resolution of two point sources^1^. Therefore, the dimer could be resolved by the cell-based lens. Because the optical image, $I\left( x, y \right)$, is the convolution of the imaging system’s point-spread function (PSF) and the object’s intensity distribution function, $O\left( \mu,\tau\right)$, it could be expressed by a standard integral equation:

$I\left( x, y \right)=\iint_{-\infty}^{\infty} O\left( \mu,\tau\right)PSF\left( \mu-\frac{x}{M},\tau-\frac{y}{M} \right)d\mu d\tau$, (1)

where *M* is the magnification of the cell-based lens. We used a Gaussian function for PSF with the full width at half maximum (FWHM) being a fitting parameter (Fig. S5c). Then, the imaging resolution was determined as the FWHM of the PSF according to Houston’s criterion, on the order of λ/5.5.


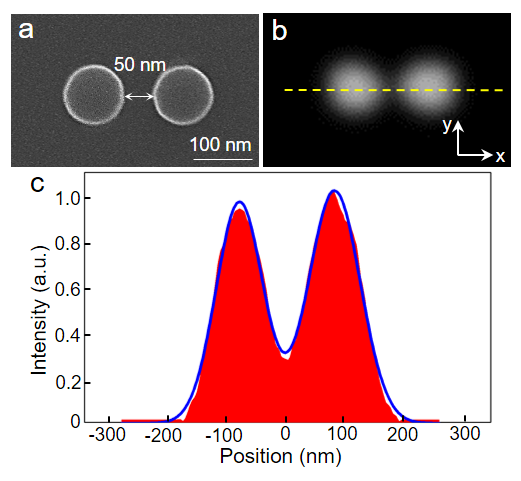


**Figure S5 | Imaging resolution measurement.** (**a**) SEM image showing a gold dimer contains two 100-nm nanocylinders with 50 nm edge-to-edge separation. (**b**) Optical image of the dimer obtained by placing a 4-μm red blood cell on the top of the sample. (**c**) Intensity profile along the *x*-axis of the dimer fitted with a Gaussian function.

**6. Imaging mechanism and resolution limit.**

To get an insight into the imaging mechanism, we have performed a simplified FEM simulation, which was basically modelled by placing two point sources at near-field region with a distance (*d*) below the cell-based lens (diameter *R*: 4 μm), as schematically shown in Fig. S6a. The imaging process could be described as that the near-field evanescence wave of nanostructures (equivalent to the point sources) converted into propagating wave through the cell-based lens, and then was collected by an objective lens in the far field and formed into an image on a detector. The imaging resolution limit was defined as the minimal distance (*s*) between two point sources that could be resolved according to Houston’s criterion. Figures S6b–d show the optical intensity distributions of two point sources with separation distance *s* of λ/3.0, λ/4.5, and λ/6.0. The black solid lines in Fig. S6b–d were drawn through the coordinate center and positions of the point sources. Figure S6e show the normalized intensity profiles at a fixed imaged plane of the two point sources with different separation distances. According to Houston’s criterion, the two point sources could be obviously resolved at a separation distance of λ/3.0, and could not be distinguished at a separation distance of λ/6.0. By changing the values of the separation distance, we found that the two point sources was exactly resolved at a separation distance of λ/4.5. Therefore, the theoretical resolution limit of the semi-immersion cell was determined as λ/4.5.


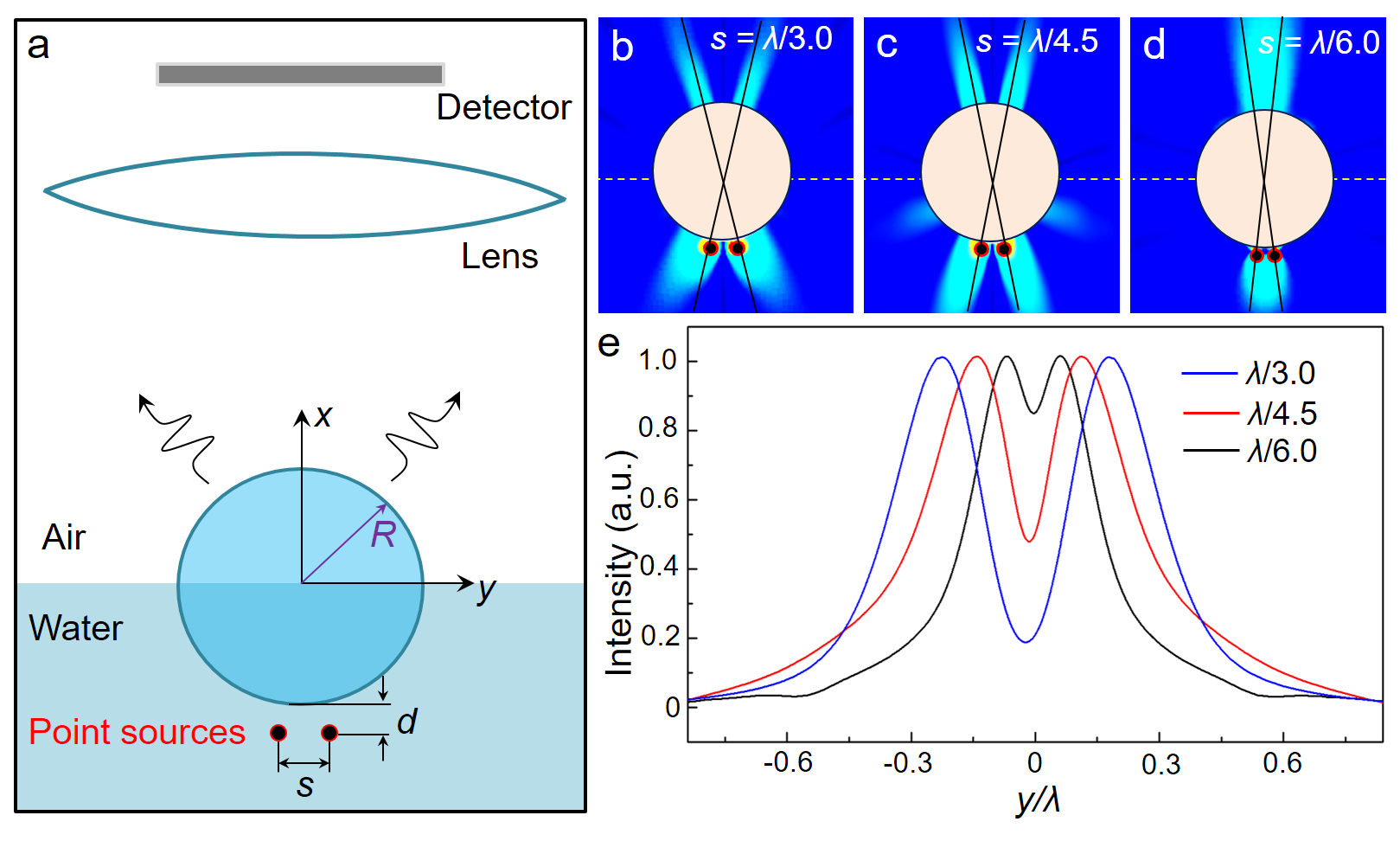


**Figure S6 | Imaging mechanism and resolution limit.** (**a**) Imaging geometry: two point sources were located below a semi-immerged cell. (**b**–**d**) Optical intensity distributions of two point sources with separation distance *s* of λ/3.0 (b), λ/4.5 (c), and λ/6.0 (d). (**e**) Normalized intensity profiles at fixed imaged plane of the two point sources with different separation distances.

**7. Temperature measurement inside the cell.**

Additional experiments have been performed to measure the temperature rise inside the cell using upconversion fluorescence nanoparticles (UCNPs). The UCNPs (NaYF_4_:Yb^3+^/Tm^3+^) with an average radius of 10 nm (inset I of Fig. S7) were used to label the cells through surface modification and endocytosis effect. Because the upconversion fluorescence was sensitive to the temperature of the medium, the UCNPs were widely used for temperature detection. As shown in inset II and III of Fig. S7, the fluorescence signals of the labelled cell before the experiment and after irradiation with an optical power of 10 mW for two hours were detected by a fiber tip coupled with a spectrometer (Ocean Optics, QE65 Pro). The average temperature of the cells could be determined by the fluorescence intensity ratio (*R*) of two different emission wavelengths, such as 450 and 650 nm. According to Boltzmann’s distribution, *R* can be expressed as^2^

$R=A\cdot e^{\frac{-\Delta E}{kT}}$ , (2)

where *A* is a constant, *k* = 1.38×10^−23^ J/K is Boltzmann’s constant, *T* is the absolute temperature, and Δ*E* = 1.36×10^−19^ J is the energy gap between the two emission wavelengths, which can be expressed as

$\Delta E=\frac{hc}{\lambda_{2}}-\frac{hc}{\lambda_{1}}$ , (3)

where *h* = 6.63×10^−34^ J·s is Planck’s constant, *c* = 3.0×10^8^ m/s is the speed of light in a vacuum, and *λ*_1_ = 650 nm and *λ*_2_ = 450 nm were the emission wavelengths, respectively. Taking the natural logarithm on both sides of Eq. (2) gives

$\mathrm{In}\left( \frac{R_{2}}{R_{1}} \right)=\frac{\Delta E}{{kT}_{1}}-\frac{\Delta E}{{kT}_{2}}$ , (4)

where *T*_1_ and *R*_1_ were the room temperature before the experiment and the corresponding intensity ratio of 450 and 650 nm emissions, which were measured as 298 K and 1.90, respectively. *T*_2_ and *R*_2_ were the temperature after irradiation and the corresponding intensity ratio, respectively. According to the fluorescence spectra in Fig. S7, *R*_2_ was measured as 2.19. Therefore, the temperature *T*_2_ was calculated as 299.3 K and thus the temperature increment Δ*T* = *T*_2_ − *T*_1_ was about 1.3 °C.


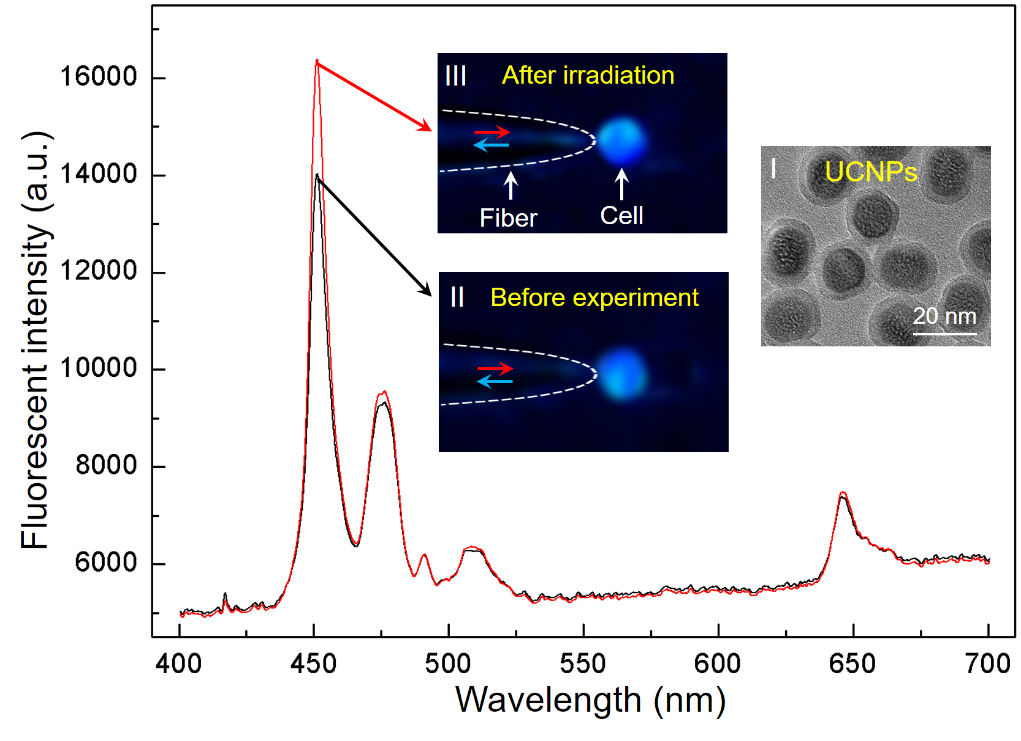


**Figure S7 | Temperature measurement of the cell.** Fluorescence spectra of the labeled cell before experiment (black curve) and after irradiation with an optical power of 10 mW for two hours (red curve). Inset I: Transmission electron microscopy image showing the UCNPs have uniformed sizes with an average radius of 10 nm. Insets II and III: Fluorescence images of the labeled cell trapped by a fiber tip before imaging experiment (II) and after irradiation (III).

**8. Cell viability tests.**

The cell viability could be observed in real time by injecting a trypan blue staining assay (Solarbio, Beijing, China) with a concentration of 0.4% into the into the cell solution with a volume ratio of ∼1:9. Dead cells would absorb trypan blue and become distinguishable from live cells^3^. As shown in Fig. S8a, the cells under irradiation with an optical power that was smaller than 45 mW for two hours were not influenced after the experiments. It means that the trapping and imaging light had no harm to the cells when the optical power was smaller than 45 mW. However, when irradiated with an optical power that was larger than 45 mW for two hours, the cells were dead and stained blue (Fig. S8b).


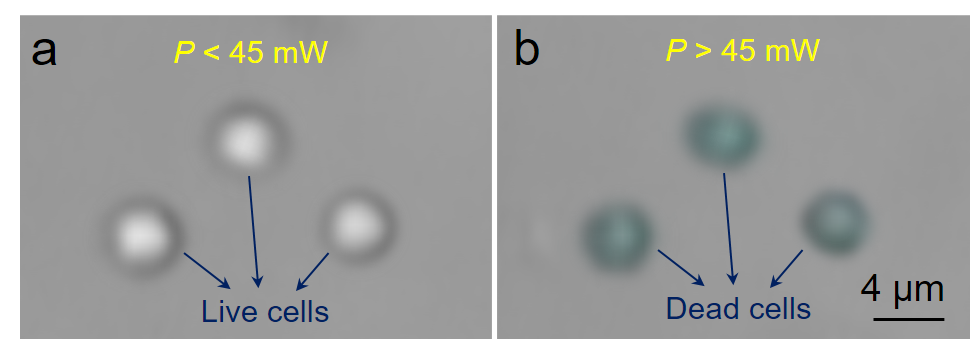


**Figure S8 | Cell viability tests.** (**a**) Optical image of the live cells under irradiation with an optical power that was smaller than 45 mW for two hours. (**b**) Optical image of the dead cells when irradiated with an optical power that was larger than 45 mW for two hours.

**References**

1. Den Dekker, A. J. & Van den Bos, A. Resolution: a survey. *J. Opt. Soc. Am. A* **14**, 547–557 (1997).
2. Vetrone, F. *et al*. Temperature sensing using fluorescent nanothermometers. *ACS Nano* **4**, 3254–3258 (2010).
3. Tan, Y. C., Hettiarachchi, K., Siu, M., Pan, Y. R. & Lee, A. P. Controlled microfluidic encapsulation of cells, proteins, and microbeads in lipid vesicles. *J. Am. Chem. Soc.* **128**, 5656–5658 (2006).
